# Profile and management of patients from low-middle socioeconomic status with thoracic trauma

**DOI:** 10.4102/sajp.v81i1.2146

**Published:** 2025-06-27

**Authors:** Heleen van Aswegen, Ronel Roos, Elizma Haarhoff, Josslyn de Kock, Humairaa Ebrahim, Sameer Tootla, Muhammad Vally, Monika Fagevik Olsén

**Affiliations:** 1 Department of Physiotherapy, Faculty of Health Sciences, University of the Witwatersrand, Johannesburg, South Africa; 2 Department of Therapeutic Services, Chris Hani Baragwanath Academic Hospital, Johannesburg, South Africa; 3 Department of Physiotherapy, Charlotte Maxeke Johannesburg Academic Hospital, Johannesburg, South Africa; 4 Department of Physiotherapy, Chris Hani Baragwanath Academic Hospital, Johannesburg, South Africa; 5 Department of Physical Therapy, Sahlgrenska University Hospital and Sahlgrenska Academy, Gothenburg University, Gothenburg, Sweden; 6 Department of Surgery, Sahlgrenska University Hospital and Sahlgrenska Academy, Gothenburg University, Gothenburg, Sweden

**Keywords:** pain, shortness of breath, range of motion exercises, thoracic trauma, physiotherapy, penetrating trauma, blunt trauma

## Abstract

**Background:**

Pain and shortness of breath (SOB) after thoracic trauma predispose patients to complications and prolonged hospital length of stay (LOS). Patient management after thoracic trauma is seldom reported.

**Objectives:**

To describe patient profiles, symptoms, management, adverse events, complications, discharge destinations and follow-up referral services.

**Method:**

Prospective observational design using clinical record review at two university-affiliated hospitals over 18 months. Adults with thoracic trauma diagnosis were consecutively screened for inclusion. Study objectives guided information retrieved from records. Statistical analyses were done with significance at *p*< 0.05.

**Results:**

Most were male (*n* = 170/179; 95%). Penetrating trauma following assault was common (*n* = 146/179; 82%). Conservative management included analgesia (*n* = 176/178; 98%) and intercostal drain insertion (*n* = 165/179; 92%). Physiotherapists treated patients daily. Management involved functional activities (cycling [*n* = 71/149; 48%], early mobilisation [*n* = 120/174; 69%]), lung volume enhancement (deep breathing exercises [*n* = 97/174; 56%], positive expiratory pressure [*n* = 98/174; 56%]), secretion removal (active coughing [*n* = 60/174; 34%]). Shoulder (*n* = 43/174; 25%) and trunk (*n* = 6/153; 4%) ROM were seldom done. Blunt trauma caused higher pain during deep breathing (median 7/10; IQR: 3.5–8.0) versus penetrating trauma (median 4/10; IQR: 2.0–7.5; *p*= 0.04). Most reported ‘slight’ to ‘very slight’ SOB. Time out-of-bed and distance walked increased daily with smokers mobilising away from bed frequently (*n* = 73/95; 77%). Few adverse events and complications occurred. Mean LOS was 5.5 ± 4.3 days. Most were discharged home (*n* = 177/179; 99%); two were referred for follow-up physiotherapy.

**Conclusion:**

Management is guided by individual patient needs. Treatment comprises early mobilisation, lung volume enhancement, and secretion removal with less attention on ROM exercises and post-discharge services.

**Clinical implications:**

Shoulder and trunk ROM should be prioritised. Service delivery approaches need review considering the evidence.

## Introduction

Thoracic injuries are frequent consequences of motor and pedestrian vehicle collisions, falls and assault globally (Dogrul et al. 2020; Edgecombe et al. 2024) and in South Africa (Skinner et al. 2015). People living in low-middle socioeconomic circumstances in South Africa are at particular risk of sustaining traumatic injury (John & Matshoba 2015). The presence of pain from thoracic trauma and reflex shortness of breath (SOB) predisposes patients to complications such as atelectasis, pneumonia and respiratory failure (Saranteas et al. 2019). Injury severity, lifestyle behaviour, age and the presence of chronic diseases are known factors that impact patients’ ability to recover from thoracic trauma (Benjamin, Nowack & Drahos 2019; Edgecombe et al. 2024; Patel et al. 2013).

In-hospital physiotherapy management of patients with thoracic trauma is not often reported in South Africa. The first reports of clinical trials on physiotherapy management of patients with penetrating stab wounds to the chest were in the 1990s by Ngubane, De Charmoy and Eales (1999) and Senekal and Eales (1994). Their participants were young adults (26–32 years) with unilateral pneumothorax, haemothorax or haemopneumothorax. The physiotherapy management approaches used included deep breathing exercises (DBE) with the physiotherapist’s hands placed unilaterally on the chest wall over the lung segments where poor ventilation was observed (e.g. lateral costal, posterior basal and diaphragmatic beathing), positive expiratory pressure (PEP) therapy in the form of balloon blowing, active coughing, active shoulder and trunk range of motion (ROM) exercises in sitting and standing, and endurance exercises such as brisk walking on the spot and around the ward, and running up and down a flight of stairs (Ngubane et al. 1999; Senekal & Eales 1994). Results showed that those who received physiotherapy immediately after intercostal drain (ICD) insertion had a shorter time to resolution of their interpleural abnormalities and shorter hospital length of stay (LOS) than when physiotherapy initiation was delayed (Ngubane et al. 1999; Senekal & Eales 1994).

Since then, no prospective data have been generated that describes physiotherapy interventions used in the acute care management of adult patients with thoracic trauma in South Africa. Similarly, no evidence exists about outcome measures used in patient management, adverse events that occur during physiotherapy sessions, patient discharge destination and referral for physiotherapy follow-up services after hospital discharge.

Firstly, our study describes patients’ characteristics, mechanisms and types of thoracic injury, clinical presentation, acute care management, adverse events, types of complications developed and discharge destination of patients from low-middle sociodemographic status admitted to participating public sector university-affiliated hospitals in Gauteng province with thoracic trauma. Secondly, it determines the difference between type of injury (e.g. blunt vs. penetrating) and levels of pain and SOB experienced at rest, and during deep breathing and functional activity, and the difference between type of injury and complications developed during hospital LOS. Smoking status and rate of mobilisation away from the bedside is also determined. Lastly, gaps in the existing structure of physiotherapy service delivery to patients with thoracic trauma are highlighted.

## Research methods and design

### Study design

A prospective observational study by means of a clinical record review was conducted. Our study forms part of a larger collaborative research project that describes management of patients with thoracic trauma between South African and Swedish trauma centres (Van Aswegen et al. 2025). In our study, the findings pertaining to the South African participating sites are presented. For this project, thoracic trauma was defined as blunt or penetrating injury resulting in pneumothorax, haemothorax, fractures of the chest wall, diaphragm and/or lung laceration, and/or pulmonary contusion (Van Aswegen et al. 2025). The STROBE checklist was used in reporting of our study.

### Setting

Patients who approached for participation were managed in the trauma intensive care unit (ICU) and wards of two public sector university-affiliated hospitals in Gauteng province. The attending trauma surgeon confirmed the thoracic trauma diagnosis. Participants were treated according to standard physiotherapy practice at the participating sites. Physiotherapy services were always available on weekdays, but the availability of physiotherapists on duty during weekends varied. During weekends, newly admitted patients or patients with acute respiratory symptoms over weekends received physiotherapy once, with standard service delivery resuming the following Monday (Van Aswegen et al. 2025).

### Study population and sampling strategy

Patients who were 18 years or older, both sexes, and diagnosed with thoracic trauma were consecutively screened for possible inclusion using the admission registers of the trauma ICUs and wards of the participating hospitals. Those with acute or previously diagnosed spinal cord injury, moderate (Glasgow coma scale (GCS) 9–12) and severe (GCS < 9) traumatic brain injury, dementia, complex pelvic fractures, lower limb fractures, lower limb amputation that restricted active mobilisation and extensive abdominal trauma (e.g. repeat laparotomy procedure or open laparotomy with drainage system) were excluded (Van Aswegen et al. 2025).

### Data collection

Meetings were held with the clinical physiotherapy staff who worked in the trauma units and wards of the two participating study sites to explain our study purposes (Van Aswegen et al. 2025). They were asked to complete participants’ details on study-specific data capture forms. The collected information included demographics (e.g. age, sex, height, weight, smoking history, presence of chronic pulmonary disease), clinical presentation (e.g. type and mechanism of injury, pain, SOB), management (e.g. medical, surgical and physiotherapy) received, complications developed during hospitalisation (Van Aswegen et al. 2025), adverse events during physiotherapy sessions (e.g. dizziness, desaturation (oxygen saturation < 92%) (Edgecombe et al. 2024), severe pain (Numeric Rating Scale score > 7/10) (Boonstra et al. 2016) and discharge information (e.g. LOS and discharge destination). The physiotherapists that oversee the trauma ICU, high care units and surgical or trauma wards at the participating sites were responsible for participant recruitment, as they screened the wards and units daily for new admissions (Van Aswegen et al. 2025). Both participating hospitals had the same protocol for data capturing and included the data prospectively. Variables covering days before participant consent were not registered (Van Aswegen et al. 2025).

All clinical physiotherapists provided the standard physiotherapy care of their institution to consenting participants. They were expected to record the specifics regarding their daily patient management and patient responses to treatment on our study-specific data capture forms. Each clinical site was visited regularly by two of the authors to meet with the clinicians and collect the completed forms to ensure an easy and simple data collection process. In these meetings, the authors reminded the clinicians not to deviate from their standard physiotherapy patient management strategies for the purposes of our study. The clinicians received training on how to use the Research Electronic Data Capture (REDCap) application on their mobile phones to capture patient information electronically; however, the participating physiotherapists at both sites felt too inexperienced with using this platform and expressed a preference for using hard copy forms. All completed forms were collected daily by the chief physiotherapists and stored in a folder in a secure area of the respective physiotherapy departments until the authors arrived for their weekly visit to collect the forms from them.

Recruitment and data collection occurred over a total of 18 months (October 2020–October 2021 at Chris Hani Baragwanath Academic hospital; August 2022–February 2023 at Charlotte Maxeke Johannesburg Academic hospital). The sample size estimation for the larger study was a minimum of 304 participants across the six participating sites (Fagevik Olsén, Sehlin & Svensson 2025) with the understanding that 50% of the sample would be collected from the two South African sites; data collection at these two South African sites was conducted over an extended period of 18 months, firstly, because of an incident at Charlotte Maxeke Johannesburg Academic hospital that delayed participant recruitment, and secondly to ensure adequate representation of South African participants within the larger study. Data were captured by a research assistant and one of the authors, for verification purposes, onto REDCap hosted at University of the Witwatersrand.

### Data analysis

Data were exported from REDCap, cleaned in Microsoft Excel and imported to IBM® SPSS® version 30 for analysis. Missing data were treated as missing. Forms with more than 50% of information not captured were excluded from analysis. Descriptive statistics were used to summarise the data. Distribution of data was tested with the Shapiro–Wilk test. Mean and standard deviation (s.d.) are reported for data with a normal distribution, alternatively median and interquartile ranges (IQR) are reported. Numbers and percentages are reported for categorical data. Comparison of findings between smoking status (yes/no) and mobilised away from the bedside (yes/no) were made using Pearson chi-squared test. To determine if the level of pain and the level of SOB experienced at rest, and during deep breathing and functional activities differed between those with blunt trauma and those with penetrating trauma, Mann–Whitney U test was performed. Pearson chi-squared test for between-group (blunt trauma and penetrating trauma) comparison was carried out for complications developed. Significance was determined at *p* < 0.05.

### Ethical considerations

Ethics approval was obtained from the University of the Witwatersrand Human Research (Medical) Ethics Committee (clearance number: M200222). Permissions were obtained from the National Department of Health, chief executive officers, trauma unit managers and physiotherapy heads-of-department at Charlotte Maxeke Johannesburg Academic and Chris Hani Baragwanath Academic hospitals in Gauteng province. The study was conducted in accordance with the Declaration of Helsinki statement as revised in 2013 to maintain participant anonymity. Written informed consent was obtained from all study participants.

## Results

**FIGURE 1:**
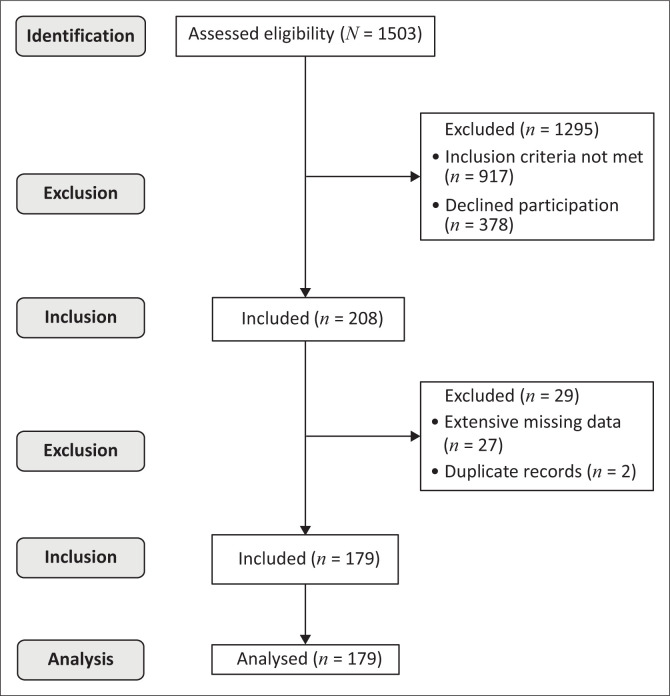
Participant recruitment and the number of participant records included in the final analysis.

A total of 1503 patients were admitted to the participating hospitals during our study period (Figure 1). After application of our study criteria, 208 patients provided consent. Twenty-nine records were excluded because of duplication or extensive missing data. The records of 179 patients were included in the final analysis, and participants’ characteristics are summarised in Table 1.

**TABLE 1:** Characteristics of participants with thoracic trauma.

Variable	Results			
	*n*	%	Mean	s.d.
Age (years)	179	-	33.0	10.0
**Sex**	179	-	-	-
Male	170	95.0	-	-
Female	9	5.0	-	-
**BMI (kg/m^2^)**	154	-	22.7	4.8
Underweight: < 18.5	23	15.0	-	-
Normal weight: 18.5–24.9	94	61.0	-	-
Overweight: 25–29.9	25	16.0	-	-
Obesity: > 30	12	8.0	-	-
Smoker†	96	64.0	-	-
Chronic respiratory disease prior to admission†	1	0.6	-	-
Length of stay (days)	179	-	5.5	4.3

BMI, body mass index; s.d., standard deviation.

† , *N* = 151.

Participants were in their mid-thirties, many had normal body mass index and most were male. The majority were current smokers with no history of chronic pulmonary disease, and mean hospital LOS was less than 1 week.

**FIGURE 2:**
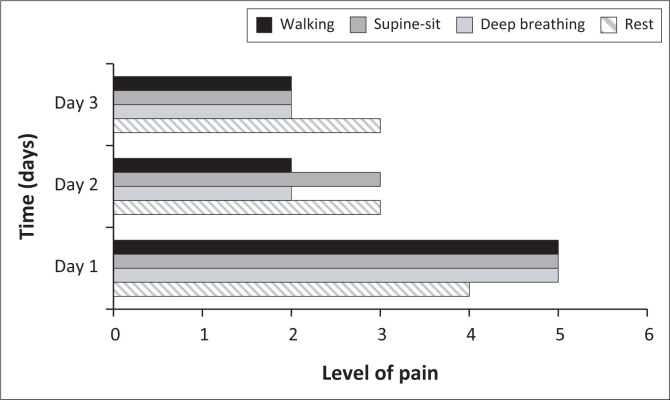
Pain (median) experienced during Day 1 – Day 3 of admission as reported by participants using the numeric pain rating or faces pain scales (0–10).

The types and mechanisms of injury sustained are presented in Table 2 and Figure 2.

**TABLE 2:** Type of injuries sustained (*N* = 179).

Variables	Results	
	*n*	%
**Type of injury**		
Blunt injury	33	18.0
Penetrating injury	146	82.0
**Rib fractures**		
None	150	84.0
1–3 ribs fractured left chest wall	8	4.5
1–3 ribs fractured right chest wall	7	4.0
4–6 ribs fractured left chest wall	5	3.0
4–6 ribs fractured right chest wall	7	4.0
> 3 ribs fractured bilaterally	2	1.0
**Intrapleural abnormality**		
None	6	3.0
Pneumothorax unilateral	41	23.0
Haemopneumothorax unilateral	122	68.0
Haemopneumothorax bilateral	10	6.0
Pulmonary contusion†	9	5.0
Lung laceration	6	3.0
Diaphragm rupture	4	2.0
Orthopaedic injury	19	11.0
Internal organ injury	23	13.0

† , *N* = 178.

The main mechanism of injury was assault (*n* = 161/176; 91%), followed by motor vehicle accidents (*n* = 9/176; 5%), pedestrian vehicle accidents (*n* = 5/176; 3%) and attempted suicide (*n* = 1/176; 0.6%). Most participants sustained penetrating thoracic trauma that resulted in unilateral haemopneumothorax. Most participants had no rib fractures; furthermore, lung laceration, pulmonary contusion and diaphragm rupture were rare. Some participants sustained orthopaedic injury external to the thorax (e.g. upper limb fractures (*n* = 9/19; 47%), facial fractures (*n* = 4/19; 21%), soft tissue injury (*n* = 2/19; 11%), lower limb fracture (*n* = 1/19; 5%) and stable spinal fracture (*n* = 1/19; 5%)). Those who sustained internal organ injury had liver (*n* = 9/23; 39%), heart (*n* = 6/23; 26%), spleen (*n* = 3/23; 13%), abdomen and/or kidney (*n* = 2/23; 9%, respectively), and large intestines (*n* = 1/23; 4%) involvement.

Table 3 summarises the medical and surgical management received by participants as recorded at the time of their first physiotherapy session.

**TABLE 3:** Medical and surgical management received by participants recorded at the time of their first physiotherapy session (*N* = 179).

Management	Results	
	*n*	%
Analgesia†	176	99
Sedation†	6	3
Mechanical ventilation	7	4
High-flow nasal oxygen	3	2
Oxygen therapy via nasal cannulae or face mask	86	48
Intercostal drainage system	165	92
Other surgery received	24	13

† , *N* = 178.

Most participants received analgesia and were managed with ICD systems. Few received mechanical ventilation (MV), high-flow nasal oxygen or sedation therapy. None of the participants were managed with noninvasive positive pressure ventilation as recorded at their first physiotherapy assessment. More than half of the participants were breathing spontaneously on room air. Some participants received surgical interventions (e.g. sternotomy (*n* = 9/24; 38%), laparotomy (*n* = 8/24; 33%), thoracotomy (*n* = 4/24; 17%) and soft tissue debridement (*n* = 3/24; 13%). None had rib fixation surgery.

Most participants received physiotherapy treatment during the first 3 days of hospitalisation, as summarised in Table 4.

**TABLE 4:** Physiotherapy interventions used to manage patients’ impairments, activity limitations and participation restrictions (Day 1 – Day 3).

Interventions	Results											
	Day 1 (*n* = 174)				Day 2 (*n* = 153)				Day 3 (*n* = 113)			
	*n*	%	Mean	s.d.	*n*	%	Mean	s.d.	*n*	%	Mean	s.d.
**Respiratory impairments**												
ACBT	80	46.0	-	-	54	35.0	-	-	35	31.0	-	-
DBE	97	56.0	-	-	82	54.0	-	-	59	52.0	-	-
Active coughing	60	34.0	-	-	40	40.0	-	-	16	14.0	-	-
Breath stacking	7	4.0	-	-	5	3.0	-	-	1	1.0	-	-
FET	5	3.0	-	-	3	2.0	-	-	2	2.0	-	-
Manual chest therapy	3	2.0	-	-	2	1.0	-	-	2	2.0	-	-
Incentive spirometry	1	0.6	-	-	1	0.6	-	-	0	0.0	-	-
PEP	98	56.0	-	-	73	48.0	-	-	49	43.0	-	-
**Musculoskeletal impairments**												
ROM	43	25.0	-	-	31	20.0	-	-	19	17.0	-	-
Trunk ROM	4	2.0	-	-	6	4.0	-	-	4	4.0	-	-
Strengthening exercises	3	1.7	-	-	1	1.0	-	-	0	0.0	-	-
**Limitations in endurance** †												
Cycling	69	40.0	-	-	71	48.0	-	-	47	44.0	-	-
Stair climbing	24	14.0	-	-	26	17.0	-	-	8	8.0	-	-
**Limitations in functional activities**												
Exercises in bed only	14	8.0	-	-	6	4.0	-	-	4	4.0	-	-
Exercises by the bedside	16	9.0	-	-	7	5.0	-	-	5	4.0	-	-
Mobilisation away from the bedside	120	69.0	-	-	103	67.0	-	-	71	63.0	-	-
**Results**												
Time sat out-of-bed in the chair (h)	-	-	1.5	1.46	-	-	2.9	2.0	-	-	3.7	2.8
Distance mobilised (m)	-	-	79.0	60.50	-	-	133.0	114.0	-	-	162.0	146.0

ACBT, active cycle of breathing technique; DBE, deep breathing exercises; FET, forced expiratory technique; PEP, positive expiratory pressure; ROM, range of motion.

† , Day 1: *n* = 173; Day 2: *n* = 149; Day 3: *n* = 106.

Physiotherapists at the participating sites did not provide treatment according to a formal care pathway but according to individual patient needs. Most participants received one physiotherapy session per day, with few (0.6% – 1%) receiving two sessions. None of the physiotherapists reported using interventions such as postural drainage, suctioning, manual hyperinflation, ventilator hyperinflation, mechanical insufflation–exsufflation, intermittent positive pressure breathing or inspiratory muscle training in their patient management. Interventions such as DBE, active cycle of breathing technique (ACBT), PEP therapy and active coughing were frequently used to address patients’ respiratory system impairments. Trunk ROM exercises and muscle strength training were seldom used for musculoskeletal system management. Functional exercises such as mobilisation away from the bedside and cycling on a stationary bicycle were commonly used treatment interventions. Sixty per cent of nonsmokers (*n* = 31/52) mobilised away from their bedside on Day 1 of physiotherapy treatment compared to 77% of smokers (*n* = 73/95). This was a significant result (*p* = 0.028).

The 2-min step test was used by most physiotherapists (*n* = 119/179; 67%) to assess patients’ response to exercise training with only one using the 6-min walk test. Time spent out-of-bed and distance mobilised gradually increased during the first 3 days of physiotherapy management. Few adverse events occurred during physiotherapy sessions. These included dizziness (*n* = 4/179; 2%), desaturation (*n* = 1/179; 0.6%), ICD malfunction (disconnection of ICD tubing from bottle) (*n* = 1/179; 0.6%) and severe pain (*n* = 1/179; 0.6%).

The levels of pain that participants experienced over the first 3 days are summarised in Figure 2.

Deep breathing and functional activities were painful for participants on Day 1; however, the levels of pain reported decreased by Day 3. Participants with blunt chest trauma reported significantly higher levels of pain on Day 1 during deep breathing (median 7/10 [IQR: 3.5–8]) compared to those with penetrating trauma (median 4/10 [IQR: 2–7.5]; *p* = 0.04). No other significant between-group differences in pain experienced were found. Physiotherapists used the faces pain scale most often for pain assessment (*n* = 119/178; 67%).

**FIGURE 3:**
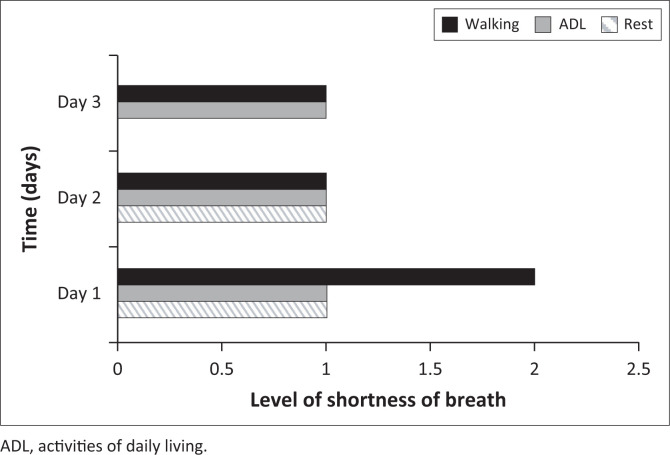
Shortness of breath (median) experienced during Day 1 – Day 3 of admission as reported by participants using the modified Borg dyspnoea scale (0–10).

Figure 3 summarises the levels of SOB that participants reported during the first 3 days of hospitalisation.

Participants reported ‘slight’ SOB during walking, which decreased to ‘very slight’ on Day 2 and Day 3. By Day 3, they had no SOB at rest. No significant differences in SOB were found between those with blunt thoracic trauma and those with penetrating thoracic trauma. Most physiotherapists assessed SOB using the Modified Borg dyspnoea scale (*n* = 164/178; 92%).

Few participants developed pulmonary complications during hospitalisation. Complications included atelectasis (*n* = 4/179; 2%) and respiratory failure necessitating MV (*n* = 1/179; 0.6%). No participant developed pneumonia. None of the participants with blunt thoracic trauma developed atelectasis versus four participants with penetrating trauma who did develop atelectasis. This finding was not significant (*p* = 0.34). The only participant who developed respiratory failure and needed MV was in the blunt chest trauma group.

Discharge from physiotherapy services occurred at the same time as discharge from the hospital for 72% of participants (*n* = 129/179). Many participants were discharged home (*n* = 177/179; 99%) from the participating sites, with the remaining 1% transferred to other hospitals. Only two participants were given a follow-up appointment for physiotherapy at the hospital outpatient department.

## Discussion

This prospective observational study provides a mirror of actual clinical in-hospital management and the normal flow of patients with thoracic trauma from low-middle socioeconomic status admitted to the two participating hospitals. Previous physiotherapy reports from Gauteng and KwaZulu-Natal are over 25 years old (Ngubane et al. 1999; Senekal & Eales 1994). Most of the current participants were male, of working age, smokers and reported no chronic respiratory diseases before the traumatic event. Assault was the most reported mechanism of injury and resulted in penetrating thoracic trauma for most participants, of which unilateral haemopneumothorax was most common. Although evidence suggests that the leading cause of traumatic injury is road traffic accidents (World Health Organisation [WHO] 2023), other South African researchers reported assault as the leading cause of thoracic trauma in similar study settings to ours (Bhana, Fru & Plani 2022; Moeng, Makhadi & Molewa 2023). This supports the notion that the types of thoracic trauma encountered are influenced by the socioeconomic status of the community within which research is conducted (GBD Collaborators 2018; WHO 2024). Additional injuries sustained, such as upper limb and facial fractures and internal organ injuries, are consistent with physical assault or assault with a weapon. The injury severity, although not formally assessed, was low for our study cohort as few required sedation, intubation and MV or noninvasive positive pressure ventilation, and few had lung laceration or pulmonary contusion. It is known that being young, male and of low socioeconomic status increases the risk of injury from physical violence (WHO 2024). Previous reports from South Africa on adults admitted with traumatic injury to the thorax show a male incidence of 92% – 95% due to men being more likely to be involved in risky behaviour than females (Bhana et al. 2022; Moeng et al. 2023). The patient demographic (e.g. age, sex, type of trauma, diagnoses) in our study conforms with these recent reports and older reports from South Africa (Ngubane et al. 1999; Senekal & Eales 1994).

Mobilisation of patients away from the bedside was the most used functional activity. Early mobilisation of patients with rib fractures who do not require MV is recommended (Battle et al. 2023). More than 65% of patients (*n* = 120/174) were mobilised away from the bedside on Day 1 in our study, which resonates with this recommendation. Participants seemed to tolerate this intervention as their time spent sitting out-of-bed in a chair and the distance they walked increased from Day 1 – Day 3 of hospitalisation. An interesting observation was that smokers mobilised away from the bedside more often than nonsmokers from Day 1 of hospitalisation. This conforms with the notion that smokers are more likely to participate in early mobilisation after sustaining thoracic trauma than nonsmokers (Battle 2020). This speaks to the smoker’s paradox, which may seem to be to the detriment of the patient but can serve as motivation to attain positive clinical outcomes.

In the first 3 days of hospitalisation, most participants were managed with ACBT or DBE, PEP therapy, and active coughing to prevent the onset of respiratory complications. The frequency of use of these interventions was reduced from days one to three. Similar treatment strategies were reported in the clinical trials by Ngubane et al. (1999) and Senekal and Eales (1994). An international multidisciplinary expert panel recommends that treatment to enhance lung volumes and facilitate secretion removal for patients with rib fractures should commence within 24 h of patient presentation to the emergency department (Battle et al. 2023). Current practice by physiotherapists at the participating hospitals resonates with existing evidence and underscores the importance of using the abovementioned interventions to prevent patients from developing respiratory complications.

Cycling on a stationary bicycle and general ROM exercises were also used in patient management. Few participants received trunk ROM exercises as part of their physiotherapy management. This is in contrast to the reports by Ngubane et al. (1999) and Senekal and Eales (1994), where all patients received combined shoulder and trunk ROM exercises (e.g. forward flexion, rotation and side-flexion) in sitting and standing to encourage drainage of blood from the interpleural spaces into their ICD bottles. Such exercises, commenced by participants shortly after admission, resulted in a shorter duration of resolution of haemopneumothoraces and hospital LOS. These authors, however, did not report the levels of pain or SOB experienced by their participants (Ngubane et al. 1999; Senekal & Eales 1994). Participants in our study reported moderate pain levels during deep breathing and functional activities, which decreased to mild pain by Day 3 of hospitalisation. All participants received analgesia, and their levels of SOB ranged from ‘slight’ to ‘very slight’ during activity. The intercostal nerves are irritated by the presence of ICD tubes and blood in the interpleural space, leading to inflammation and pain (Mergner 2017). All study participants by the abovementioned authors had ICD tubes *in situ* and would have experienced similar levels of discomfort.

Some participants in our study underwent surgery (e.g. sternotomy, laparotomy, etc.). Surgery is a known cause of disruption of the myofascial structures and severe pain, especially during deep breathing, coughing and movement (Kolettas et al. 2015). In contrast, the studies by Ngubane et al. (1999) and Senekal and Eales (1994) did not include patients who underwent surgery. Physiotherapists who participated in a global survey on the management of adults with major thoracic trauma indicated that they frequently used active shoulder girdle (72%) and trunk ROM (62%) exercises for patient management (Van Aswegen et al. 2020). Furthermore, the expert recommendations published by Battle et al. (2023) emphasise the commencement of bilateral upper limb and shoulder ROM exercises from admission and within limits of pain for patients with rib fractures and no concurrent upper limb injury. From the available data, it is unclear whether physiotherapists at the participating sites considered shoulder and trunk ROM exercises to assist blood drainage from the interpleural spaces to enhance the resolution of their patients’ haemopneumothoraces. They may have felt that such exercises would be too uncomfortable for their patients to perform after sustaining thoracic trauma.

Physiotherapists used outcome measures such as the 2-min step test, numeric pain rating, faces pain scale and the modified Borg dyspnoea scale during their management. The numeric pain rating and face pain scales recognise patient self-report measures of pain in trauma populations, as is the modified Borg scale as a self-report measure of SOB experienced (Morrow, Wright & Van Aswegen 2024). The 2-min step test is listed as one of the outcome measures used by physiotherapists who work in trauma settings on a global level to assist with physiotherapy discharge planning for adults with thoracic trauma (Van Aswegen et al. 2020). It is, therefore, reasonable to conclude that the choice of outcome measures used in physiotherapy patient care in our study reflects evidence-based practice.

Physiotherapy service delivery over weekends, public holidays and in early intervention areas such as the emergency department is greatly impacted by human resource constraints at the participating hospitals. Physiotherapy commenced in the trauma ward or ICU during working hours. Participants mostly received one physiotherapy session per day. This contrasts with the frequency of treatment reported by Ngubane et al. (1999) and Senekal and Eales (1994), where some participants received physiotherapy treatment in the trauma ward immediately after the insertion of their ICD system. These authors showed that those who received immediate physiotherapy intervention had better outcomes relating to ICD drainage time, hospital LOS and development of complications than those who received physiotherapy 9–24 h after ICD insertion. Participants in our study had a mean hospital LOS of 5.5 days, which is longer than that reported by Senekal and Eales (1994) (mean 3.2 days) and shorter than that reported by Ngubane et al. (1999) (mean 7.5 days) for their participants who received delayed initiation of physiotherapy services. There is still something to be said about the timing of treatment after insertion of ICD, which was not tracked in terms of hours in our study. This is something that could guide future clinical practice in the setting. It is possible that the COVID-19 pandemic, during which our study was conducted, impacted shortening patients’ LOS. Physiotherapy service was not delivered according to a clinical care pathway at the participating sites. The variability in the treatment participants received could be attributed to a patient’s presentation and assessment findings or to the resources they had access to in their place of work.

Desaturation, dizziness, severe pain and ICD malfunction were reported for a small number of participants in our study during physiotherapy sessions. These were all temporary in nature and completely resolved. Atelectasis and respiratory failure necessitating intubation and MV were the only complications that developed for a small number of patients during hospitalisation. In contrast, empyema was reported by Ngubane et al. (1999) and spiking temperatures were reported by Senekal and Eales (1994). Risk factors for the development of empyema include thoracic trauma and pneumonia (Garvia & Paul 2024). None of our study participants developed pneumonia, which might explain why empyema was not observed in this cohort during a hospital stay. The onset of symptoms of empyema takes up to 15 days (Garvia & Paul 2024). It is possible that some participants could have developed empyema after hospital discharge. Monitoring of patient readmissions to the participating hospitals because of empyema was beyond the scope of our study.

Two participants were transferred to another hospital and the rest were all discharged to their homes. Again, it is possible that the COVID-19 pandemic impacted patients’ discharge destinations. Only two participants were given a referral to attend outpatient physiotherapy after discharge. This low rate of referral of patients recovering from thoracic trauma to outpatient physiotherapy services is comparable to the 8% of physiotherapists who referred their patients for outpatient follow-up in the global survey (Van Aswegen et al. 2020). It is known that conservatively managed rib fractures lead to chronic pain and SOB, resulting in long-term respiratory disability after discharge and negatively impact health-related quality of life, in some cases up to 11 years after injury (Carrie et al. 2019; Kelderman et al. 2024). Factors influencing physiotherapists’ decision to refer or not refer patients for postdischarge follow-up need exploration. Recording of patients’ postdischarge outcomes, such as the prevalence of chronic pain symptoms and health-related quality of life, is required to inform the development of a clinical care pathway.

Our study had some limitations. Admission rates of patients with thoracic trauma to the two hospitals were impacted by the various levels of lockdown experienced in South Africa during the COVID-19 pandemic. No injury severity data were directly retrievable from patient files at participating hospitals. The prospective nature of our study could have led to bias regarding patient consent for capturing information from their records for study purposes. However, the procedures dictated by the ethics committee that approved our study require that researchers also request written consent from patients to include their information in retrospective record review designs. Data regarding physiotherapy interventions used were self-reported, which may have led to bias in capturing the data or changes in clinical practice by clinician participants to conform with what they perceived the researchers wanted for study purposes. There was a large amount of missing data related to physiotherapy management from day four onwards; hence, management could only be objectively reported for Day 1 – Day 3. The services of external research assistants who could visit the participating hospitals daily during the data collection period and capture study-specific data from patient records would have been beneficial to limit the amount of missing data and to ensure that physiotherapy patient management was not altered for study purposes. Such persons could not be employed for our study because of limited available funds. The study was conducted at two public sector university-affiliated hospitals in Gauteng province, which limits the interpretation and generalisation of findings in other trauma settings.

### Recommendations

Future research should focus on in-depth exploration of physiotherapy service delivery to patients with thoracic trauma through clinical audits to expose factors that impact service provision to such patients in public sector university-affiliated hospitals. Private healthcare settings where patients with thoracic trauma are frequently managed should be included in future studies. The effect of the inclusion of shoulder and trunk ROM exercises in physiotherapy management of adults with thoracic trauma on their clinical outcomes, such as days to ICD removal, hospital LOS and incidence of pulmonary complications, needs renewed attention through experimental research. Engagement with members of the local trauma teams is encouraged to facilitate the development of clinical care pathways that are appropriate for the patient demographics seen at the respective hospitals.

## Conclusion

The findings of this study demonstrate that translation of evidence into clinical physiotherapy practice is evident through the strategies employed by clinicians at the participating hospitals to prevent the onset of respiratory complications, to prioritise early mobilisation, and in their selection of measurement tools to assess patient outcomes, for adult patients with thoracic trauma. It draws attention to shortfalls in clinical practice related to musculoskeletal physiotherapy rehabilitation and physiotherapy service delivery during weekends.
